# Development, Testing and Results of a Patient Medication Experience Documentation Tool for Use in Comprehensive Medication Management Services

**DOI:** 10.3390/pharmacy7020071

**Published:** 2019-06-20

**Authors:** Stephanie Redmond, Nicole Paterson, Sarah J. Shoemaker-Hunt, Djenane Ramalho-de-Oliveira

**Affiliations:** 1 Ridgeview Medical Center, Waconia, MN 55387, USA; Stephanie.Redmond@ridgeviewmedical.org; 2 Fairview Pharmacy Services, Medication Therapy Management Services, Minneapolis, MN 55414, USA; npaterson@fairview.org; 3 Abt Associates, Cambridge, MA 02138-1168, USA; Sarah_Shoemaker@abtassoc.com; 4 Center for Pharmaceutical Studies, College of Pharmacy, Universidade Federal de Minas Gerais, Belo Horizonte 31.270-901, Brazil

**Keywords:** comprehensive medication management services, medication experience, pharmaceutical care practice, documentation, focus groups

## Abstract

The medication experience is an individual’s subjective experience of taking a medication in daily life and can be at the root of drug therapy problems. It is recommended that the patient-centered approach to comprehensive medication management (CMM) starts with an understanding of the patient’s medication experience. This study aims to develop a medication experience documentation tool for use in CMM services, and to understand the usefulness and challenges of using the tool in practice. The tool was developed based on previous research on patients’ medication experiences. It was tested in two rounds by ten CMM pharmacists utilizing the tool as they provided care to patients. Focus groups were conducted to revise the tool after each round and to understand pharmacists’ experiences. The tool was tested for 15 weeks in 407 patient encounters. There was at least one medication experience documented in the electronic medical record 62% of the time. Pharmacists found the tool helpful in raising awareness of the medication experience and motivational interviewing strategies, planning for follow-up visits, as a teaching tool, and making pharmacists realize the fluidity of the medication experience. The tool offered pharmacists a better way to recognize and address medication experiences affecting medication taking behaviors.

## 1. Introduction

The profession of pharmacy continues to shift from the traditional dispensing role to a more patient-focused practice revolving around the provision of Comprehensive Medication Management (CMM) services. Several studies have shown the value of CMM in the care of patients with chronic conditions through the resolution of drug therapy problems [1,2,3,4]. It should be noted that there are different approaches to medication management services emerging in practice: the prescription-focused and the patient-centered approach. The first one is related to activities performed at the time of dispensing a drug to a patient. The patient-centered approach delivers the service on an appointment basis applying specific standards of care to each patient encounter [4].

In providing a patient-focused service, CMM pharmacists have been challenged with using evidence-based medication guidelines, while understanding the patient’s unique medication experience to improve patient outcomes [5,6,7]. The medication experience is an individual’s subjective experience of taking a medication in daily life and includes the patient’s preferences, feelings, concerns, beliefs, and behaviors associated with medications [5]. Patients often have perceptions and beliefs about medications based on their own experiences or the experiences of others, which may influence or even prevent them from taking medications as recommended [5]. Patients might adjust doses to minimize unwanted effects and make the regimen more acceptable, or they can weigh risks against benefits, as a response to perceptions that go from gratitude that medicines exist to fear and uncertainty about adverse effects, or they can consider two or more medications being used for the same condition as unnecessary, or they might even completely deny their illness [8]. Other concerns that patients may have include fear of the actual administration of medicine or drug dependence. These concerns are often due to insufficient knowledge of the medication [8]. In this context, understanding the meanings that medications have for patients may help pharmacists to positively impact their medication-taking behavior [5].

Previous research has shown that pharmacists have used the patient’s medication experience to guide the education provided to patients starting new medications to prevent the development of drug therapy problems (DTP), as well as to tailor their interventions to resolve drug therapy problems [6]. Ultimately, research findings indicate that there are many examples of drug therapy problems for which the medication experience is at the root cause [7].

In order to improve these experiences or change patients’ perceptions of their medications, different strategies can be utilized in practice. Some studies show that strategies to overcome a negative medication experience are focused around influencing a perception, an attitude or a behavior change [8,9,10]. This is not always as simple as resolving a DTP, where a dose can be adjusted or a new medication started, if another one was not effective. Motivational Interviewing (MI) has been found to be an effective intervention to promote behavioral changes. It is a form of collaborative conversation between provider and patient directed towards strengthening a patient’s own motivation and commitment to change. MI strategies are particularly useful for those who are reluctant or ambivalent about changing a behavior [11,12,13]. Also, some research has indicated that CMM pharmacists have successfully used MI strategies to affect patients’ medication experiences [6,7].

It is recommended that the patient-centered approach to comprehensive medication management starts with an understanding of the patient’s medication experience [4,14]. However, documenting the patient’s medication experience and intentionally using this knowledge to make decisions about strategies to resolve DTPs and meet patients’ medication-related needs is still not a common habit in practice.

The purpose of this research was to develop a medication experience documentation tool for use in CMM services, test the tool in practice with experienced CMM pharmacists, and understand the usefulness and challenges of having pharmacists use the tool in practice.

## 2. Materials and Methods

**Figure 1:**
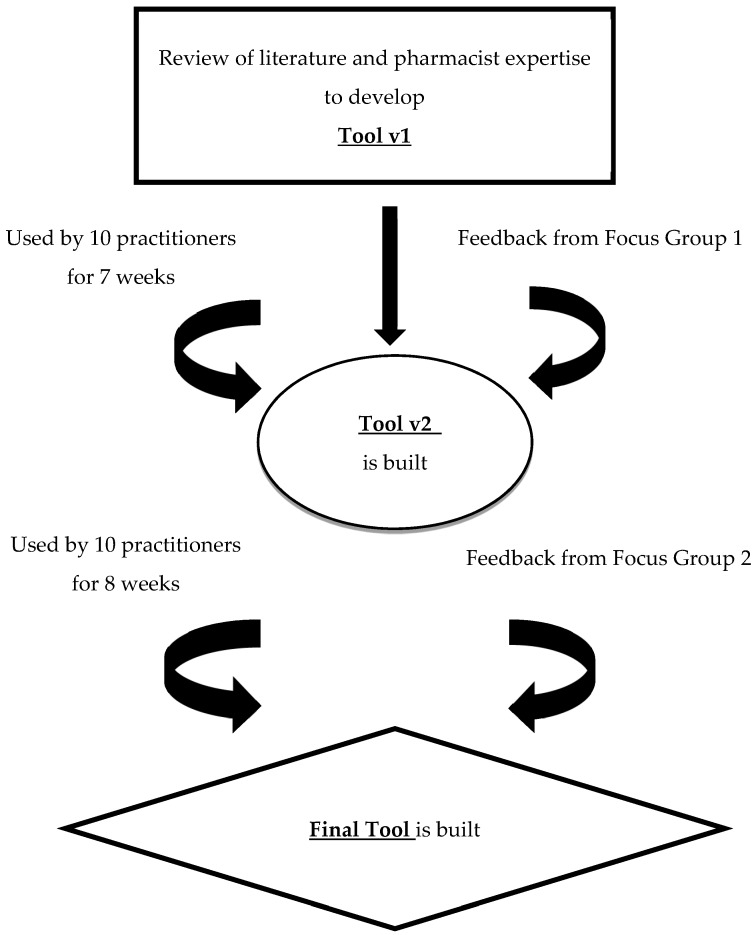
Approach to Developing and Testing the Medication Experience Documentation Tool.

The tool was iteratively developed and refined based on two rounds of testing in practice and focus groups with CMM pharmacists. The methods are described by tool development, testing, and focus groups, as seen in Figure 1.

### 2.1. Tool Development

The aim of the medication experience documentation tool was to facilitate the documentation of a patient’s medication experiences perceived to be at the root of drug therapy problems (DTPs) by CMM pharmacists, as they conducted an assessment of a patient’s medication-related needs. The tool also allows pharmacists to indicate the motivational interviewing strategies used to address the identified medication experience and associated DTPs. 

The initial version of the medication experience assessment tool (Tool v1) was developed based on previously published research on patients’ medication experiences and pharmacists’ perspectives encountering patients’ medication experiences in their practice [3,4,5,6,7,14] and motivational interviewing strategies [11,12,13]. The tool was built in the electronic medical record (EMR), where CMM pharmacists routinely document the care they provide. The tool included two drop-down lists from which to select: (1) the patient’s medication experience and (2) the motivational interviewing strategies used to address that specific medication experience. There was also a write-in option for any experience or strategy not reflected in the list and a ‘none’ option if no experience was identified at that specific visit. The tool was located at the end of the assessment section of the EMR documentation. The pharmacists were instructed to use the tool during every patient encounter, though only medication experiences that negatively impacted a patient’s medication-taking behavior were deemed to require an intervention and thus be documented with the tool. Multiple medication experiences could be identified during a single visit, thus the drop-down boxes allowed for multiple options to be selected. However, the pharmacist would document only the experiences he or she attempted to affect during that specific encounter.

### 2.2. Tool Testing

#### 2.2.1. Setting

The tool was tested with pharmacists in a large health care system consisting of 7 hospitals and 38 ambulatory clinics. CMM services have been provided in this health system for over 15 years. Participating pharmacists had a range of experience practicing CMM spanning from 4 months to 15 years and were all practicing in ambulatory primary care clinics. CMM pharmacists document their progress notes in the electronic medical record in the form of a SOAP note (subjective/objective/assessment/plan). All of the CMM pharmacists participating in this study had previously undergone training on how to use the medication experience tool and on motivational interviewing. In practice, the pharmacists identified medication experiences through the pharmaceutical care assessment process by asking open-ended questions and listening for conversation clues. Then, motivational interviewing techniques were used to affect those experiences that were perceived by the pharmacist as negatively impacting the patient’s medication taking behavior. They were instructed to use the documentation tool during the time that they conducted the assessment of each patient.

#### 2.2.2. Testing Period

All 20 CMM pharmacists practicing in the health care system were invited to contribute to this research and 10 agreed to participate. The participating ten pharmacists utilized the first version of the tool (Tool v1) for seven weeks as part of their practice providing comprehensive patient-centered medication management (CMM) services to patients in the primary care setting. The same 10 pharmacists used the second version of the tool (Tool v2) for eight (8) weeks.

#### 2.2.3. Tool Results

The EMR of all patients cared for by the 10 participating pharmacists during the studied period were analyzed to identify the type and the number of experiences documented as well as the strategies utilized to address those experiences in all patient encounters. All data were documented in an excel spreadsheet and the frequencies of the identified medication experiences and the utilized strategies were calculated.

### 2.3. Focus Groups with Pharmacists

Two focus groups were conducted with all participating ten pharmacists using the techniques proposed by Krueger and Casey [15]. The aims of the focus groups were to understand pharmacists’ experiences using the documentation tool in practice, in terms of usefulness and challenges.

A focus group was conducted after each testing period with Tool v1 and Tool v2. The information revealed in the focus groups informed revisions to the tool. The focus groups were two hours in length. A focus group discussion guide did not used any specific framework, but it was developed with the purpose of stimulating discussion to elucidate pharmacists’ experiences, reactions, and expectations related to the medication experience and motivational interviewing strategies in the tool. See Table 1 for a list of questions used in the focus groups.

All focus groups were audio-recorded and then professionally transcribed verbatim. All researchers independently coded the data to identify significant sections about pharmacists’ perspectives on the tool. Then, they met to form a consensus about the common themes of pharmacists’ experiences and perspectives on utilizing the documentation tool. The final themes are described below.

#### Human Subjects Protection

Pharmacists were assured confidentiality that any information provided could not be traced to them or to individual patients. This study was approved by the Institutional Review Board at the University of Minnesota.

## 3. Results

### 3.1. Tool Development

After the completion of testing Tool v1 and the first focus group, several changes were incorporated into Tool v2, see Table 2. After the completion of testing Tool v2 and the second focus group, changes were also incorporated into the final tool, see Table 2. The final tool can be found in Appendix A.

### 3.2. Tool Utilization and Results

A summary of the tool testing approach, utilization and results is provided in Table 2. In round 1, pharmacists utilized the tool 29% of the time in 180 of 620 patient encounters in the 7-week testing period. In round 2, pharmacists utilized the tool 35% of the time in 227 of 649 patient encounters in the 8-week testing period. The tool was used 32% of the time in a total of 407 patient encounters. 

When the tool was used in 407 patient encounters, it was observed that patients had a documented medication experience 62% of the time. Specifically, in round 2, no medication experience was selected in 29%, 86 out of 292 total medication experiences. Table 3 shows round 2, the most common medication experiences identified and the most common strategies utilized by the pharmacists to address each medication experience; presented in decreasing order of frequency. Definitions used for the strategies can be found in the Appendix B.

### 3.3. Pharmacist Focus Groups

The major themes that emerged from the focus groups are described below in terms of the usefulness and challenges of using the medication experience documentation tool. The findings reflect the experiences of the 10 CMM pharmacists that utilized the tool and participated in both focus groups.

#### 3.3.1. Usefulness of the Tool

Participants expressed the usefulness of the tool as the following themes: the tool makes CMM pharmacists more aware of the medication experience and more reflexive; using the tool makes pharmacists realize the fluidity of the medication experience; the tool assists with teaching; and the tool helps with future care plans.

##### The Tool Makes CMM Pharmacists More Aware of the Medication Experience and More Reflexive

The group of 10 CMM pharmacists expressed feelings that the tool led them to document more details of the visit in relation to the medication experience and the reasoning behind a patient’s medication taking behaviors. Some of them asserted that usually they use the tool as an afterthought. They also expressed that the documentation process changed their practices, as they felt more conscious of and more likely to address and explore a patient’s medication experience to help patients to better understand the need for their medications and what to expect from them.

“The fact that I had to think about the experience and what strategies I would use, it changed my practice. I put it into an important piece of my every day work. …and then I started thinking I should really use this strategy the next time I talk to a patient about this type of thing” (Pharmacist 3).

Awareness was created and the pharmacists felt more able to truly identify what the patient wants out of the visit.

“I feel more aware and I am thinking about it more. Before it was more subconscious” (Pharmacist 1).

Using the tool and documenting the medication experience helped CMM pharmacists to become more reflexive. As the pharmacist in the last quote emphasized, it was felt that documenting the medication experience helped them to be more aware of patients’ experiences and what they could do to improve those experiences.

Since the tool was embedded into note templates, during the CMM visit, pharmacists could see it on the computer screen. It was agreed that simply seeing the tool during the visit was a good reminder and created consciousness of MI strategies that could be used during the consultation to motivate the patient.

“When I see it [the tool] there in the visit, it causes me to think about it with the patient and make sure I get to the bottom of it” (Pharmacist 8).

##### Using the Tool Makes Pharmacists Realize the Fluidity of the Medication Experience

As participants documented the medication experience at each visit, it became more evident how fluid and dynamic these experiences can be.

“The same patient can have different experiences associated with different medications” (Pharmacist 4).

“The experience can change from one visit to the next. So, we really need to pay attention to the patients’ expressions at each visit. We cannot predict what is happening today based on our last visit” (Pharmacist 1).

To understand the changeability of the medication experience was an important result of utilizing the tool. These experiences are subjective and impacted by different factors meaning that the pharmacist needs to carefully listen at each visit and use the appropriate strategies to address them when needed.

##### The Tool Assists with Teaching

Participants in the focus group agreed that one of the most useful aspects of the documentation tool was for teaching purposes—for both students and new CMM pharmacists. It helps to frame concepts that CMM pharmacists are naturally evaluating, but are not necessarily able to explain or discuss aloud with students or novice pharmacists.

“When I am with a student, the tool pushes me to express what is inside my head, the connections I am making to take a certain decision” (Pharmacist 10).

“The medication experience is a difficult concept to teach because people tend to think that is too philosophical, too abstract. But it is definitely not! The tool helps to bring the medication experience to a more practical level when I am teaching” (Pharmacist 2).

Several pharmacists emphasized how using the tool helped students to pay more attention to the subjective aspects of taking medications, which is usually a type of knowledge neglected in pharmacy curricula.

“By using it [the tool] students start to become more attentive to what kinds of factors impact patients’ decisions and behaviors regarding their meds” (Pharmacist 5).

##### The Tool Helps with Future Care Plans

Documenting the medication experience was found to be helpful for deciding a course of action for the next patient’s visit.

“It was helpful for follow-up visits to know how to approach that patient next time if you knew how they were feeling and how they responded to certain strategies… It helps me know what avenue to go down to or to avoid based on previous visits.” (Pharmacist 4)

“I tell my student, after looking at a previous note [with an experience documented in it], that we have to frame our next conversation with the patient considering this information” (Pharmacist 9).

#### 3.3.2. Challenges of Using the Tool

Participants revealed the challenges of the tool as the following themes: being reflexive can be burdensome and unearthing the difference between the medication experience and a drug therapy problem.

##### Being Reflexive Can Be Burdensome

The CMM pharmacists felt they were already subconsciously identifying and addressing the medication experience during their routine assessment of a patient, so to have to stop and document forced them to reflect, which was described at times as “burdensome”. It was often difficult to categorize experiences since this is a component of the assessment that the group was not used to documenting.

“I think it is just hard to stop and reflect on how patients’ feelings and previous experiences with medications can impact their attitudes and decisions. I know this is there, it’s everywhere, but to document it forces me to rethink over and over again” (Pharmacist 1).

“It takes time. So, it can be challenging” (Pharmacist 6).

It should be noted that this setting includes a group of experienced CMM pharmacists—all with experience in MI and having prior understanding and exposure to the medication experience concept.

“The tool may be more redundant for us at times because our group does this innately, but it would probably be even more helpful for other groups” (Pharmacist 10).

##### Unearthing the Difference between the Medication Experience and a Drug Therapy Problem

For pharmacists, one of the most important challenges in using this tool was to understand the difference between a medication experience and a DTP, since at times they seem to overlap. It was agreed that the medication experience is completely subjective and usually emotional—typically revolving around a strong feeling or fear. It describes the patient’s relationship with medications and provides the background on how it changes their views on medications, and often times their behaviors. It is not present in all patients.

For several participants in the study, using the documentation tool multiple times with many different patients triggered them to perceive the difference between a DTP and a medication experience.

“Before I started using the tool I never really stopped to think about how the medication experience is the same or different from a DTP. I felt there was a connection, but now I know they are related but different concepts” (Pharmacist 5).

The group ultimately defined a negative medication experience as “when an experience can get in the way of a patient getting the most benefit from a medication and can lead to a drug therapy problem”.

“To me, the medication experience is associated with feelings or attitudes toward taking medications that is getting in the way of doing what I think is best for them” (Pharmacist 7).

A patient could have multiple DTPs that are not necessarily associated with a medication experience. On the other hand, a patient might have a medication experience, but no DTP identified at that point in time.

## 4. Discussion

The results showed that using the tool has utility for the practice of patient-centered comprehensive medication management services. Even though it can be “overwhelming” or burdensome to document one more aspect of the patient care process, the course of documenting the patient’s medication experience makes the CMM pharmacist more reflexive. Reflexivity has been shown to be an important trait of a patient-centered practitioner [16,17]. Reflexivity assists pharmacists to become more present at consultations, more attentive to patients’ needs and more open to patients’ unique perspectives and experiences. In addition, reflexivity may help pharmacists to look more critically at themselves as professionals and human beings, which creates space for a broader awareness regarding their way of relating to patients and other health care providers [18].

Almost all of the participating pharmacists reported using the documentation tool as an after-thought. Yet some used it to stimulate ideas of motivational interviewing during the encounter, so having it embedded into the note template was fundamental in making it useful. Interestingly, most of the strategies listed in Table 3 are the same regardless of the medication experience identified. This may suggest that some strategies are more effective in general or possibly easier to use. On the other hand, it may reflect that pharmacists may not be familiar with certain MI strategies and implies that further work is needed to learn and implement new interventions in practice. Similarly, in adherence studies that utilized motivational interviewing, no one intervention consistently enhanced adherence for all patients alike because many variables affect a patient’s decision-making process. Typically, a combination of interventions is needed to best address a patient’s needs [19]. As MI has outperformed traditional advice given for a broad range of behavioral problems and diseases, having pharmacists refine these skills may be a useful way to impact patient health outcomes [20].

For the participants in this study, using the tool helped them to emphasize to students the importance of the medication experience in patients’ decision-making and, therefore, in the delivery of CMM services. Students often do not realize that many patients do not take their medications without resistance and naturally tend to focus much more on their knowledge of pharmacotherapy versus understanding their patients’ experiences and goals. Using the tool to document the medication experience helps bring a broader understanding to that concept. The pharmacists remarked that they had seen it shape their students’ approach to patients’ visits and create attentiveness to what types of factors impact patients’ decisions and behaviors regarding medications. In other words, the documentation tool might have encouraged pharmacists and students to become more patient-centered. As indicated by previous research, incorporating patients’ medication experiences into clinical decision-making is not an easy task, thus, it is paramount to teach pharmacy students and novice pharmacists how to identify and use this information in daily clinical practice [6,7,20,21,22].

Another important outcome of using the tool to document patients’ medication experience was a better understanding of the difference between a drug therapy problem and a medication experience. For instance, consider the DTP that a patient needs additional drug therapy. There is no need to have a patient experience involved with it unless there is resistance to starting the medication, which would need to be further explored by the pharmacist. An example of a DTP not correlated with a medication experience would be when a dose adjustment is needed, and the patient’s perceptions or feelings are not associated with this. A caveat to this would be if the patient refuses to increase the dose because he or she fears side effects or if they equate taking a higher dose with worsening health status or personal failure, which reflects a medication experience. The CMM pharmacists agreed that a DTP may or may not be linked to an experience or attitude towards taking a medication. However, when that is the case, it is the CMM responsibility to address this experience with the goal to improve it or include the patient’s perspective into the decision-making process [20].

Several situations emerged with utilizing the medication experience documentation tool. The medication experience was found to be dynamic and often changed from visit to visit. Therefore, the CMM pharmacists agreed they should be intentional in assessing and documenting the presence of a medication experiences at every single visit. The CMM pharmacists also expressed that it was ultimately important to document if, at a particular visit, a patient did *not* have a medication experience that was affecting medication taking or patient outcomes—hence, the creation of the ‘none’ option for medication experience. It was agreed that the most meaningful way to use the tool was to limit the selection of strategies to a maximum of the one or two that were the most effective that day. This helped them to avoid becoming “desensitized”, as it was hard to interpret which strategies were most effective when preparing for a follow-up visit if several strategies were listed.

The research recently conducted by Nascimento et al. corroborates the results of this study as it exposed the complexity of the medication experience. It emphasized the fact that the same individual can simultaneously experience daily medication use in diverse ways, depending on the medical condition and the medication used. In that study, patients experienced daily medication use in four general ways: resolution, adversity ambiguity, or irrelevance. These ways are related to the manner the medication affects the patient´s personal world, which means that taking a medication is much more than purely a mechanical action. Medications can disturb the patient´s relationship with his body, her perception of herself, or his relationship with others [22].

Certain logistics of the tool were challenging with regard to placement of the tool in a documentation note, understanding when to use the tool, and keeping the documentation brief and simple enough for convenience and ease of use. The group could not troubleshoot how to prevent from misinterpreting that a medication experience applies to all medications a patient is taking versus just one. The tool was not linked to certain conditions or medications. The experience can change based on the medication and can be different for each visit, so the challenge moving forward is understanding how to link the documented experience to a specific medication without making the tool too lengthy and cumbersome to use.

It should be noted that this setting includes a group of experienced CMM pharmacists—all with experience in MI and having some prior understanding and exposure to the medication experience concept. As suggested by other studies, educational programs would likely be needed to teach how to identify patients’ medication experiences and documenting them with the tool and how to apply MI strategies to their practice [21,22].

It is important to state that the use of the documentation tool in the patient’s assessment has become the standard of practice for CMM pharmacists in the studied health care system. There needs to be an expansion of available strategies to understand the best way to improve a patient’s medication experience. Evaluating positive medication experiences may also provide insight on effective methods and strategies to use with patients. Limited published information shows the use of the pharmacist applying MI as a strategy to address the medication experience. There are studies that show trends of improved medication adherence when MI is used as an intervention by nurses, physicians, and therapists in patients with chronic conditions [23]. Improved therapy adherence is associated with lowered healthcare cost and increase in workplace productivity [24]. Our study looked much further than adherence, as the medication experience can impact all categories of drug therapy problems (indication, effectiveness, safety, and adherence) [6,20,21]. More studies should measure how intervening on the medication experience affects specific drug therapy problems and patient outcomes.

### Limitations

This study should be interpreted in light of its limitations. The initial tool was developed based on studies that aimed for a patient-centered understanding of patients’ medication experiences and pharmacists’ experiences encountering these experiences in practice.

During the testing time period, there was a substantial gap between the total number of patient encounters and the encounters in which the tool was used resulting in selection bias. Possible reasons for this could be that the pharmacists simply forgot, were too busy to use the tool in their documentation, or did not recognize a medication experience important enough to document. It took time for pharmacists to incorporate the documentation tool into their existing practices. While MI has been shown to outperform traditional advice giving in the treatment of a broad range of behavioral problems, no new strategies were identified during the focus group and the group of pharmacists using the tool struggled with utilizing the listed strategies [20]. Additionally, the design of the documentation tool did not identify if a strategy was effective or not. It documented that a certain strategy was used during the visit, but the effectiveness of the strategy was not followed up on at the subsequent visit to determine true effectiveness of the intervention. Only negative medication experiences were assessed with this tool, as we assumed these to be the most meaningful to impact and require intervention for improved outcomes.

The results originated from a group of CMM pharmacists within one health system, and can not necessarily be extrapolated or generalized to other practices.

## 5. Conclusions

CMM pharmacists commonly encounter patients’ medication experiences in their practices. The documentation tool was developed and tested to assist pharmacists to better recognize patients´ medication experiences and elect associated strategies to improve medication outcomes. Pharmacists’ acknowledged that awareness of the medication experience and a toolbox of strategies to affect it helped improve patient-centered care in their practices. Continued reflection and documentation of the medication experience is essential in continuing to advance CMM and pharmacist patient-centered practice.

## Figures and Tables

**Table 1:** Focus Group Discussion Questions.

Focus Group 1	Focus Group 2
What was it like for you to document your patients’ medication experiences? ○ Has it been helpful to you to document these experiences, how and why? What medication experiences or strategies should stay, should not be there, what are missing? How do you see this being useful tool for other practitioners, new practitioners, students? Has documenting the medication experience been useful for follow-up visits?	
What do you think of the tool? What was it like to use the tool?	Is the revised tool easier to use? What would make it easier and/or more helpful?
Can the tool be used in patients with no medication experiences?	Is this tool important enough to incorporate as a requirement in our documentation notes?
	Is the placement of the tool in the “assessment” section appropriate?
	Does documenting and having a more in-depth awareness of patient’s medication experience help with or change your therapy decisions?
	Has this helped you in developing your motivational interviewing skills? How?
	Are you noticing the medication experience can change over time?
	In your experience with documenting these patients’ experiences, how do they connect to drug therapy problems?

**Table 2:** The Medication Experience Documentation Tool: Overview of Testing Rounds, Use and Changes.

	Round 1	Round 2
**Tool Version**	Tool v1	Tool v2
**Testing Duration**	7 weeks	8 weeks
**Pharmacists**	10 RPhs	10 RPhs (same as Round1)
**Pharmacists’MTM experience**	4 months–15 years	4 months–15 years
**# of Patient encounters**	620	649
**# (%) Patient encounters pharmacists used Tool**	180 (29%)	227 (35%)
**Changes to the Tool**		
**Changes to the Medication Experiences**	- Condensed and reordered the tool into subcategories for ease of use - One medication experience deleted: ”positive medication experience, does not want to use alternative non-drug therapy or lifestyle changes” - Four medication experiences added: “does not want to use because they are too reliant on Rx medication (s)”, “feelings of burden and being overwhelmed”, “fear of side effects from personal history”, and “fear about history of addiction” - One medication experienced revised: economic value question changed to “cannot reconcile value of medication over cost”	- Shortened verbiage in tool - Moved some subcategories back into major categories - Added and removed one medication experience each - Combined one medication experience
**Changes to Strategies**	- Removed empathy and listening	- Added referral to care team member

**Table 3:** Most Common Medication Experiences Encountered and Motivational Interviewing (MI) Strategies Used for Round 2.

Most Common Medication Experiences (Med Exp)	Medication Experiences N (%)	MI Strategies Used (% for each Med Exp)					
		Raise Concern, Educate or Inform	Collaboration/SDM	Open-Ended Question	Support Autonomy	Roll With Resistance	Negotiate
Self-adjusts medication regimen (increases, decrease, or skips doses)	31 (15%)	87%	77%	77%	52%	19%	19%
**Feelings of burden and being overwhelmed (e.g., pill burden)**	27 (13%)	93%	78%	67%	56%	41%	37%
**Fears/concerns about side effects:** **History of personal side effects.**	23 (11%)	91%	87%	70%	35%	30%	26%
**Fears/concerns about side effects:** Current personal side effects.	17 (8%)	76%	82%	76%	65%	18%	35%
**Prefers alternative non-drug therapy or lifestyle changes.**	16 (8%)	81%	88%	88%	44%	6%	6%
**Fear of not having medication(s)/(security).**	10 (5%)	80%	90%	70%	60%	40%	20%
**Does not want to take medications: Does not like to take them (cultural background, other).**	10 (5%)	90%	80%	70%	50%	40%	20%
Other Experiences.	72 (35%)						
**Total**	**206 (100%)**						

Note: SDM: shared decision-making.

## Appendix A. The Medication Experience Documentation Tool

**MEDICATION EXPERIENCE AFFECTING MEDICATION OUTCOMES FOR TODAY’S VISIT** (select one or more and then select associated overall strategies):
None
Feelings of burden and being overwhelmed (ex: pill burden)
Does not want to take medications because: Doesn’t like to take (cultural background, other) Doubts the need Doesn’t feel the benefits Can’t reconcile value of medication(s) over cost Associates with a “stigma” and is ashamed/embarrassed to use it Does not like administration (swallow, infusion, injection) process
Fears/concerns about side effects: Current personal side effects History of personal side effects Observing friend or family member experience Media
Fears/concerns about becoming dependent on a medication
Fears/concerns medication (ex: generic) will not be effective
Self adjusts medication regimen (increases, decrease, or skips doses)
Increasing medication(s) or increasing medication dose(s) means: Personal failure Worsening health status
Prefers alternative non-drug therapy or lifestyle changes
Fear of not having medication(s)/too reliant on Rx medication(s) (security)
*** (write-in)
STRATEGIES USED TODAY THAT EFFECTIVELY IMPROVED MEDICATION RELATED OUTCOMES:
Open ended questions
Collaboration/Shared Decision Making
Negotiate
Evocation/Empower
Support autonomy /Emphasizing personal choice and control
Ask permission
Raise concern, educate, or inform
Reframe/ Reflection
Roll with resistance
Using a rating scale, on scale of 1-10, Importance *** Confidence ***
Elicit change talk
Encourage non-drug therapy or lifestyle changes
Referral to care team member
*** (write-in)

## Appendix B. Motivational Interviewing Strategy Definitions (11–13)

1. **Open ended questions:** Using stems such as “what” and “how” that can elicit unlimited answers from a patient and invite them to talk.
2. **Collaboration/Shared Decision Making:** Co-developing goals with the patient to make sure they are involved in creating a plan that will indeed work for the patient.
3. **Negotiate:** Meeting the patient “in the middle”. Perhaps the plan does not exactly match what you want as the practitioner, but is a smaller more reasonable goal is agreed upon that acts as a stepping stone to reach the ultimate goal.
4. **Evocation/Empower:**
  - Helping your patient explore how they can make a difference in their own health by utilizing their own ideas and resources.
  - Facilitating the patient in bringing their own expertise to the discussion on how best to accomplish change.
  - Drawing out the patient’s own motivation to change.
5. **Support autonomy/Emphasizing personal choice and control:**
  - Acknowledge to the patient that they are ultimately responsible for change and as the practitioner we separate ourselves from the outcome.
  - Communicating respect and dignity of the patient through unconditional positive regard.
6. **Ask permission:** Informing or advising only after the patient has given you permission to do so. Forms of permission would include a patient asking for advice or asking the patient for permission to inform (like knocking on a door before entering). Examples are; “May I make a suggestion?” or prefacing advice with “You can tell me what you think of this idea….”.
7. **Raise concern, educate, or inform**: The actual act of providing patient with information and advice.
8. **Reframe/Reflection:**
  - Captures and returns to patients something about what they have just said (rephrasing one or two ideas)
  - They do not have to be accurate (could be amplified or exaggerated) and may or may not introduce new meaning.
  - Different types of complex reflections: adding content or meaning, amplification, double-sided, reframing, verbalizing unspoken emotion, emphasizing one side
9. **Roll with resistance:** When patients display signs of resistance, such as blaming or minimizing, the practitioner lets the resistance flow rather than use oppositional tactics, such as telling the person he is wrong.
10. **Using a rating scale, one a scale of 1–10, Importance***Confidence***** Using a numerical rating scale or other ruler to assess the patient’s attitude toward importance and confidence behind changing a certain behavior. Typically asked as: “Could you tell me, on a scale from 1 to 10, how important it is for you to _____? Why did you give yourself a score of __ and not 1?” The same style question can be asked about confidence. Could follow up with a question such as “How can I help you move higher up the scale?” These types of questions will help support self-efficacy.
11. **Elicit change talk:**
  - Explore how their current behavior is inconsistent with their values.
  - Selectively asking and identifying when patients state desire, ability, reasons for, and the need to change and responding to this by evoking, affirming, and reflecting these back to them.

**Encourage non-drug therapy or lifestyle changes**: Supporting non-prescription therapy to help patient reach goals and outcomes.
